# Graphite to diamond transition induced by photoelectric absorption of ultraviolet photons

**DOI:** 10.1038/s41598-021-81153-3

**Published:** 2021-01-28

**Authors:** Ana I. Gómez de Castro, Maikel Rheinstädter, Patrick Clancy, Maribel Castilla, Federico de Isidro, Juan I. Larruquert, Tomas de Lis-Sánchez, James Britten, Mariona Cabero Piris, Federico P. de Isidro-Gómez

**Affiliations:** 1 U.D. Astronomía y Geodesia (Departamento de Física de la Tierra y Astrofísica), Universidad Complutense de Madrid, 28040 Madrid, Spain; 2 Joint Center for Ultraviolet Astronomy, Universidad Complutense de Madrid, 28040 Madrid, Spain; 3 Department of Physics and Astronomy, McMaster University, Hamilton, ON L8S 4M1 Canada; 4 Origins Institute, McMaster University, Hamilton, ON L8S 4M1 Canada; 5 Universidad San Pablo-CEU, Escuela Politécnica Superior, 28668 Boadilla del Monte, Spain; 6 Consejo Superior de Investigaciones Científicas, GOLD-Instituto de Optica, 28006 Madrid, Spain; 7 Instituto IMDEA Nanociencia-ICTS Centro Nacional de Microscopía Electrónica, 28040 Madrid, Spain; 8 Centro Nacional de Biotecnología, 28049 Madrid, Spain

**Keywords:** Materials science, Nanoscience and technology

## Abstract

The phase transition from graphite to diamond is an appealing object of study because of many fundamental and also, practical reasons. The out-of-plane distortions required for the transition are a good tool to understand the collective behaviour of layered materials (graphene, graphite) and the van der Waals forces. As today, two basic processes have been successfully tested to drive this transition: strong shocks and high energy femtolaser excitation. They induce it by increasing either pressure or temperature on graphite. In this work, we report a third method consisting in the irradiation of graphite with ultraviolet photons of energies above 4.4 eV. We show high resolution electron microscopy images of pyrolytic carbon evidencing the dislocation of the superficial graphitic layers after irradiation and the formation of crystallite islands within them. Electron energy loss spectroscopy of the islands show that the sp^2^ to sp^3^ hybridation transition is a surface effect. High sensitivity X-ray diffraction experiments and Raman spectroscopy confirm the formation of diamond within the islands.

## Introduction

The two most common allotropes of solid carbon are graphite and diamond. In graphite, carbon atoms are arranged in sheets, weakly bound together by van der Waals forces with an interlayer separation of ~ 3.4 Å. Within each sheet the atoms are disposed in a honeycomb lattice, each atom linked to the three neighbours through strong covalent sp^2^ bonds. In diamond, carbon atoms are bonded through sp^3^ bonds in a cubic network. The investigation of the Cañón del Diablo meteorite uncovered another sp^3^ allotrope, the lonsdaleite, where carbon atoms are bonded in a hexagonal crystalline structure^1–3^. Lonsdaleite is the less common of the two diamond polytypes and it is not detected free in nature. In meteoritic samples, it comes intermingled within the diamond network showing features consistent with faults in the diamond network^4^.

The phase transition from graphite to diamond has been extensively studied. Diamond is a highly desirable material with many applications, from abrasives and coatings to electronics, and carbon is an abundant material in nature. The transition is driven by increasing the pressure and the temperature of any kind of graphitic sample^5,6^ either by laser driven shocks^7^, or directly, by the detonation of carbon-containing explosives^8,9^. Recent experiments have resolved the dynamics of this process; it occurs in time scales of nanoseconds at working pressures that the depend on the specific type of graphite; 19 GPa in highly ordered pyrolytic graphite (HOPG) and 228 GPa in polycrystalline graphite^8^.

In the last decade, a new set of experiments have proven that the sp^2^ to sp^3^ transition can also be achieved by exciting and heating electrons in π orbitals^10–13^. Optical photons (1–4 eV) from a femtosecond laser source are directly absorbed into π–π* transitions creating a first population of hot electrons that thermalize in less than 25 fs; they achieve a Boltzmann’s statistical distribution reaching electron temperatures that may exceed 5500 K^13^. This electronic population is sufficiently hot to populate the interlayer band (energy 4.4 eV above vacuum) from where electrons may undergo thermoionic emission. The sp^2^ graphite to sp^3^ diamond phase transition seems to be caused by instabilities of the graphitic lattice induced by the redistribution of electrons from the π bands with bonding character to the interlayer band. The relevance of graphite lattice distortion for the stabilization of defects in the graphite layered structure is well known^14^. Also, the graphite layered structure may render unstable by inducing excited holes^15^.

In this work, we show that nanodiamonds are formed after the irradiation of graphitic samples (HOPG and polycrystalline graphite) by UV photons with energies above 4.4 eV. This finding provides a new and easy-to-implement method to produce nanodiamonds. It also calls for a re-evaluation of the processes to form nanodiamonds in space.

## Results

Several graphite samples (see “Materials and methods”) were irradiated in a clean, vacuum chamber. The working pressure of the chamber is 2 × 10^–3^–7 × 10^–4^ Pa (0.7 × 10^–8^–2 × 10^–8^ atm) thus, any possible contamination was removed before the samples were irradiated.

**Figure 1:**
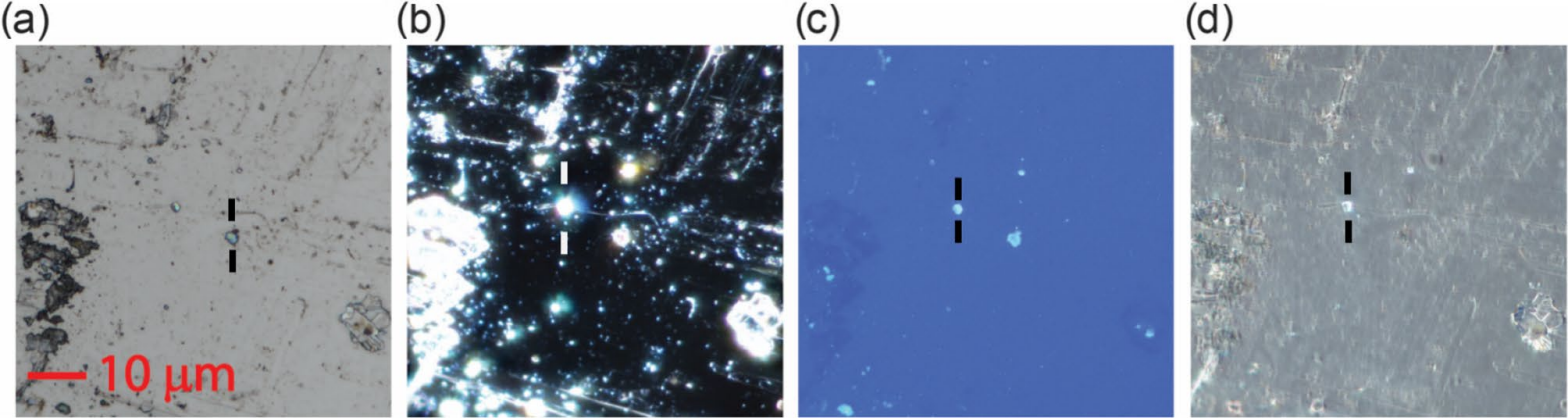
Optical microscope image of the HOPG sample after 13 h of UV irradiation: field (**a**), dark field (**b**), fluorescence (**c**) and polarization (**d**) set ups. Crystallites are formed at some locations on the surface (one of them is marked in the images). The rest remains apparently unaltered. Increasing the exposure time increases the number of crystallites formed.

After UV irradiation, featureless HOPG samples show several crystallites and structures on the surface; their sizes range from few microns to several hundredth nanometers (Fig. 1). They are neither uniform in size nor uniformly distributed and disperse white light into colours as expected for crystalline structures. The number of crystallites increases with the irradiation time.

**Figure 2:**
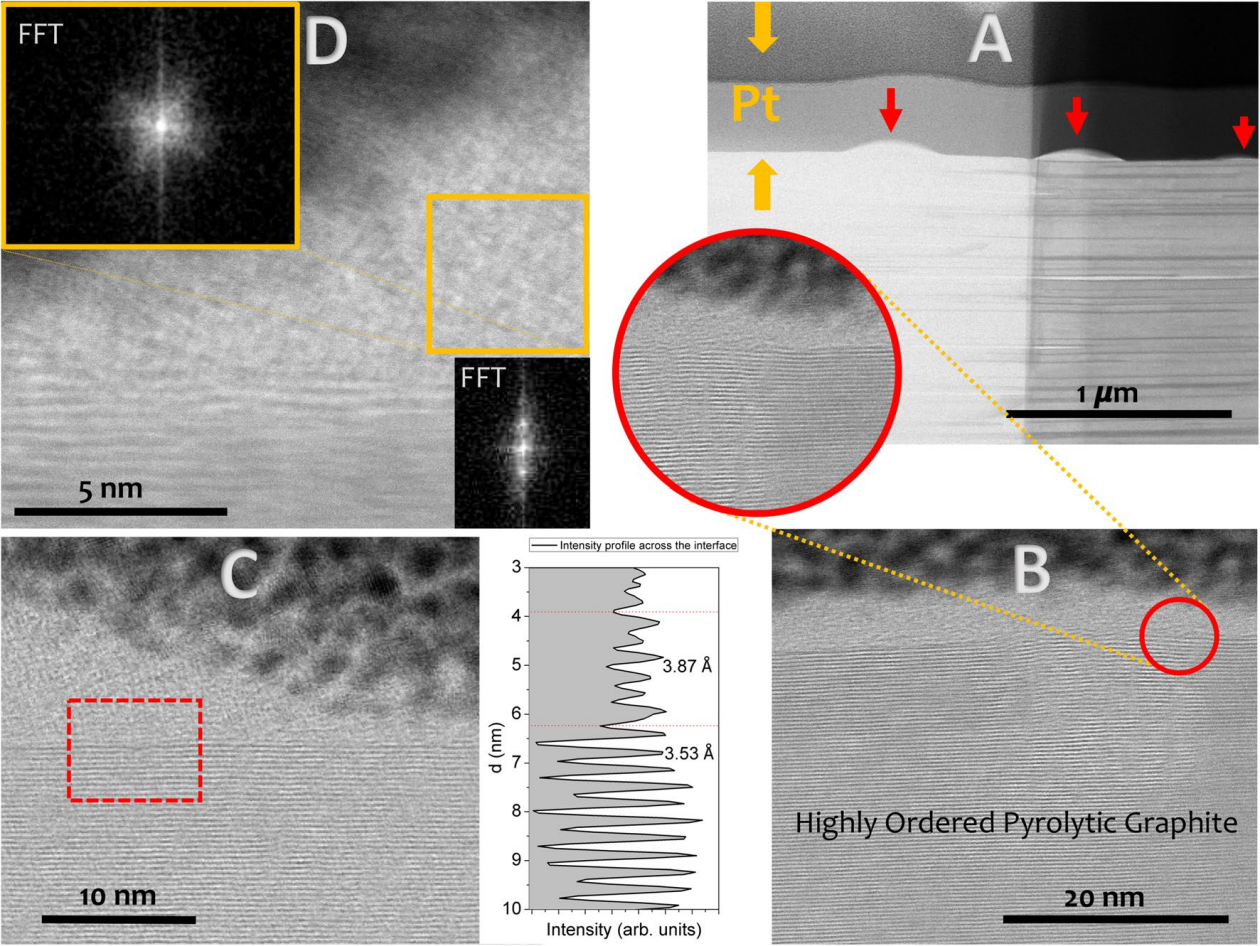
Annular bright field (ABF) images of the irradiated HOPG sample; the magnification of the images increases from (**A**) to (**D**) panels. (**A**) The low magnification image shows bumps or islands of hundreds of nm marked with a red arrow that are formed after UV irradiation of the sample (the change in the contrast is due the thickness gradient of the sample produced during FIB preparation). (**B**) High resolution ABF images show the dislocation of the HOPG layered structure at the irradiated surface (red circle). A cover a few nanometers thick forms between the HOPG and the environment. (**C**) High resolution ABF image of a bump; the separation between the graphene layers is plotted in the bottom right panel. There is a transitional area where separation between the layers increases from 3.53 to 3.87 Å. (**D**) Some apparently cubic structures form within the bump; the FFT shows the marked differences between the HOPG layered structure (bottom right inset) and the rotated patter in the bump (upper left inset).

Aberration-corrected scanning transmission electron microscopy (STEM) and EELS data have been obtained using an aberration corrected JEOL JEM ARM200cF operated at 200 kV using a condenser lens aperture of 1 mm. The high resolution annular bright field (ABF) images also show that the interlayer separation increases from 3.53 to 3.87 Å in the nearest 20 nm from the surface; in addition, some faults and dislocations are observed (see Fig. 2). The 2-D Fourier transform in the bumps is markedly different from the rest of the HOPG sample. The rotated cubic network hinted from the high-resolution STEM images neatly shows in the Fourier space and it is in clear contrast with the well-ordered and layered structure observed in the rest of the HOPG sample.

**Figure 3:**
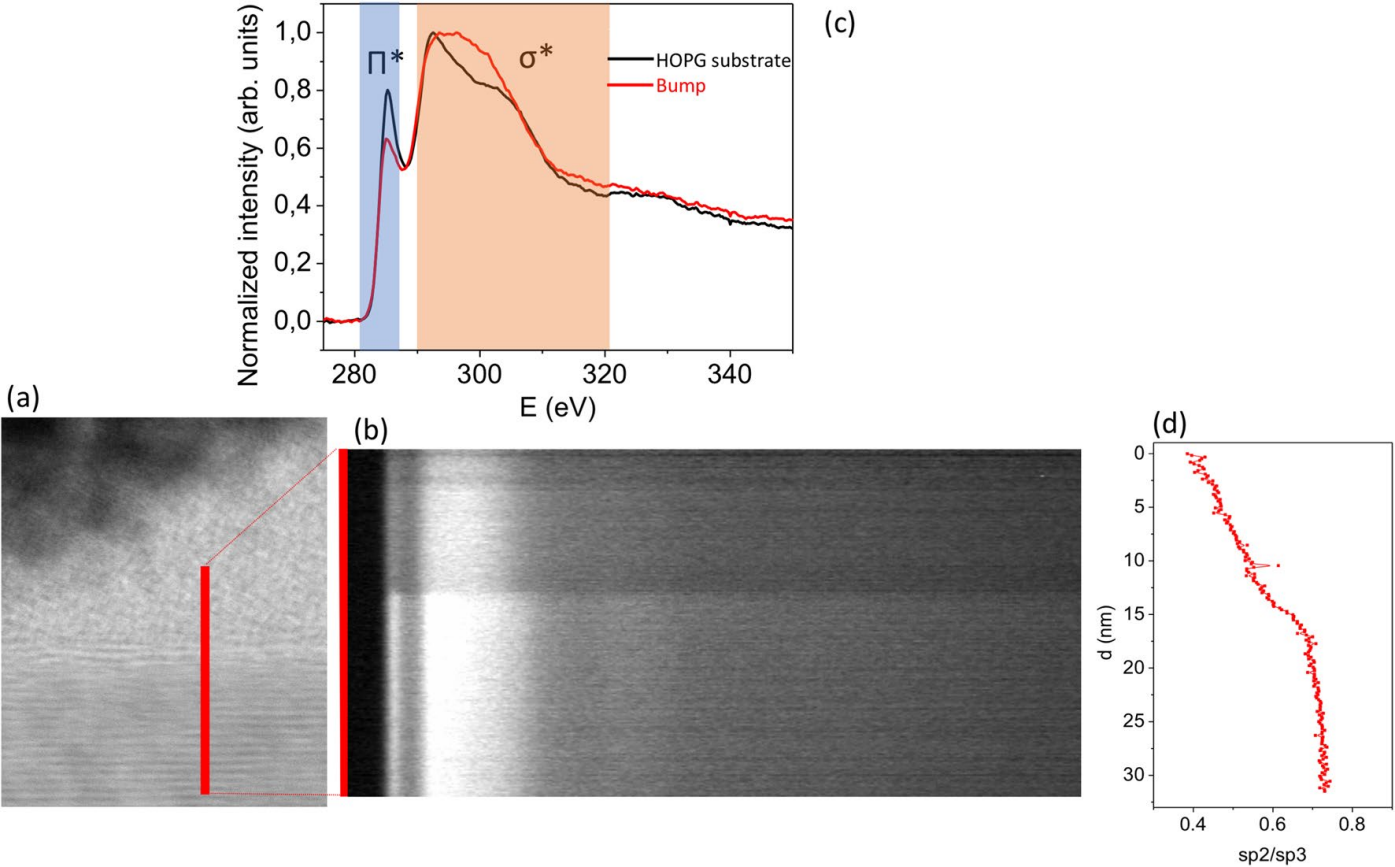
Electron energy loss spectra (EELS) of the UV processed HOPG: (**a**) Location of the slit on the material, (**b**) long-slit spectra, (**c**) averaged spectra in the HOPG substrate and in the bump. In (**c**) the location of the spectral signature of graphite’s Π* bond is marked. Note that the Π* bond signature is weaker in the bump but still present, meaning that the bump is not pure diamond. The ratio of the signal in the Π* and σ* spectral bands (**c**) is used to define the sp^2^/sp^3^ index that it is displayed in (**d**). The signal in the Π* spectral band is minimal on the surface and increases inwards.

Electron energy loss spectroscopy spectroscopy (EELS) confirms that there is a transition in the structural properties. The trigonal bonding of C atoms in graphite can be detected by the signatures of the Π* and σ* bonds at 282–288 eV and 290–320 eV however, no Π* bond exists in diamond. EELs spectra show that the relative strength of Π* with respect to σ* bonds decreases significantly in the bumps when compared with the HOPG substrate however, it does not vanish pointing out that the phase transition does not occur over the full volume. The sp^2^/sp^3^ rate increases from the surface to the interior of the sample (see Fig. 3).

HOPG samples also have been analyzed using Auger spectroscopy providing similar results. Measurements were made on the bumps, as well as, on the apparently unaffected surface. Diamond and graphite Auger spectra are clearly distinct and their respective character can be determined by fitting the Auger spectra from any sample area. The highest diamond fraction of 35% was found on a bump, and the lowest diamond fraction was measured to be 12.8% on the substrate area. All measurements run on the substrate detect diamond though in a small fraction (~ 13%).

To further examine the composition of the bumps, we carried out high flux X-ray diffraction measurements using the A2 beamline at the Cornell High Energy Synchrotron Source (CHESS). Instead of using the irradiated HOPG samples, we irradiated carbon fibres for this experiment. There are two reasons for that; firstly, both electron microscopy and Auger experiments on HOPGS showed that the phase transition occurs within a layer of a few hundred nanometres thickness hence, any potential signal from diamond would be diluted in the noise of the strong graphite signature in the 0.6 mm thickness HOPG sample. Moreover, the number of photoelectrons ejected per absorbed UV photon is likely to be significantly greater for the small graphite crystallites within the carbon fibres than in the large planar surfaces of HOPG thus enhancing the impact of UV radiation in structural changes; note that photon absorption occurs closer to the surface in small particles thus, photoelectrons have a better chance to escape^16,17^.

A collection of UV irradiated fibres was mounted on the goniometer of a Huber four-circle diffractometer. Measurements were carried out in vertical scattering geometry, using a high efficiency 2D area detector (Pilatus 300 K). The energy of the incident X-ray beam was tuned to E_i_ = 14.5 keV (λ = 0.8551 Å).

A series of X-ray pole figures were collected at scattering angles (2θ) in the vicinity of the first two *diffraction (Bragg) peaks* expected for the diamond crystal structure: (1, 1, 1) and (2, 2, 0). These pole figures allow us to survey large volumes of reciprocal space, integrating the number of X-rays scattered over a narrow range of momentum transfers (Q), and providing an effective method of searching for the characteristic diffraction signature from diamond. This approach to structural characterization is required due to the small size of the UV-induced crystallites, and the relatively weak X-ray form factor associated with carbon (Z = 6).

**Figure 4:**
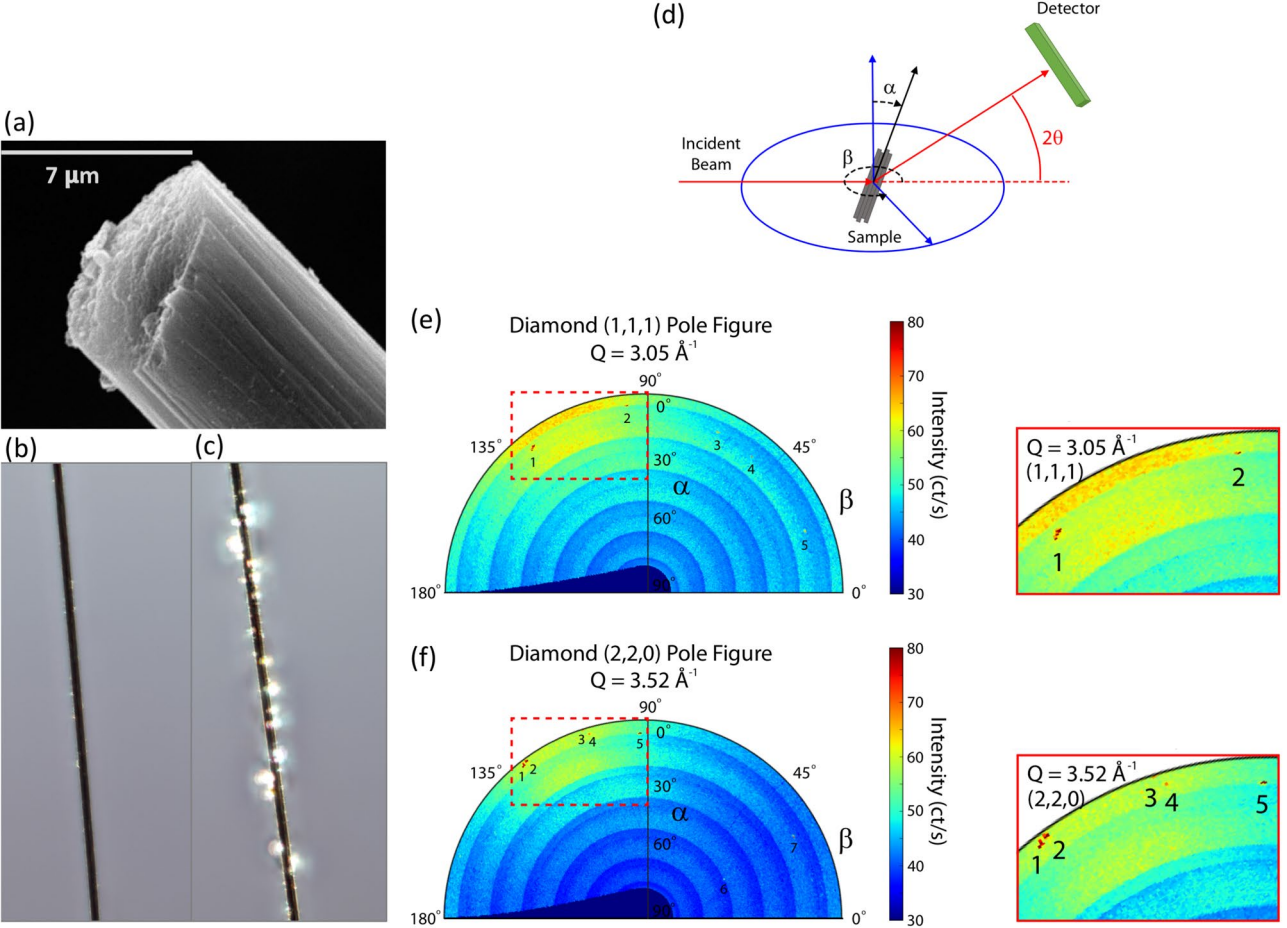
(**a**–**c**) images of carbon fibres: (**a**) high resolution image obtained with the electron microscope JSM 6400 from the National Centre of Microscopy of Spain; (**b**) optical  microscope image of a fibre before irradiation and (**c**) after irradiation. The results from the X-ray diffraction measurements with CHESS are displayed in (**d**–**f**): (**d**) schematic drawing of the experimental geometry; (**e**) X-ray pole figure collected at the scattering angle corresponding to the diamond (1, 1, 1) Bragg peak [Q = 3.035–3.065 Å^−1^]; (**f**) X-ray pole figure collected at the scattering angle corresponding to the diamond (2, 2, 0) Bragg peak [Q = 3.50–3.54 Å^−1^]. The existence of well-defined Bragg peaks in (**e**) and (**f**) provides strong evidence of crystalline diamond in the irradiated fibres.

As shown in Fig. 4, the X-ray pole figures reveal evidence of sharp, well defined, diffraction peaks at scattering angles corresponding to both the (1, 1, 1) and (2, 2, 0) Bragg peaks from crystalline diamond in the irradiated fibre samples. We observe about 5–7 distinct peaks in each pole figure, indicating the presence of multiple crystallites within the sample. The relatively narrow angular width of these peaks (~ 0.5° full width half maximum) indicates a high degree of crystallinity.

It should also be noted that these peaks are quite weak, with intensities ~ 200 times weaker than the observed diffraction peaks corresponding to the graphite structure of the fibres. This appears to be consistent with the size and volume fraction of the crystalline defects observed in the optical microscope images.

**Figure 5:**
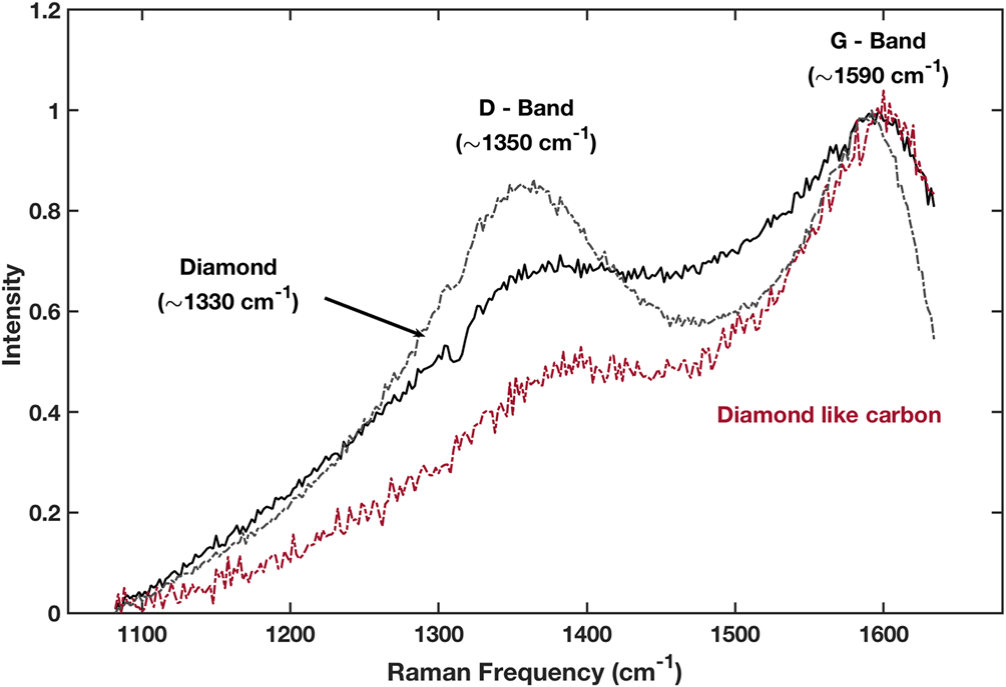
Calculated electron yield from graphite caused by the irradiation with the commercial L10366 Hamamatsu Deuterium lamp. The calculations have been made using the known yield from bulk graphite which is the least favourable case^20^.

In addition, we have obtained Raman spectra of the crystallites that confirm the formation of diamond. The spectrum shown in Fig. 5, displays the two prominent bands observed in carbonaceous materials: D band at ~ 1350 cm^−1^ and G band at ~ 1590 cm^−1^. The G-band results from the stretching of the C–C bond in graphitic materials and is common to all sp^2^ carbon systems. The D-band results from disorders in sp^2^-hybridized carbon systems^18^ and it is blended with the diamond feature at ~ 1330 cm^−1^^19^.

## Discussion

**Figure 6:**
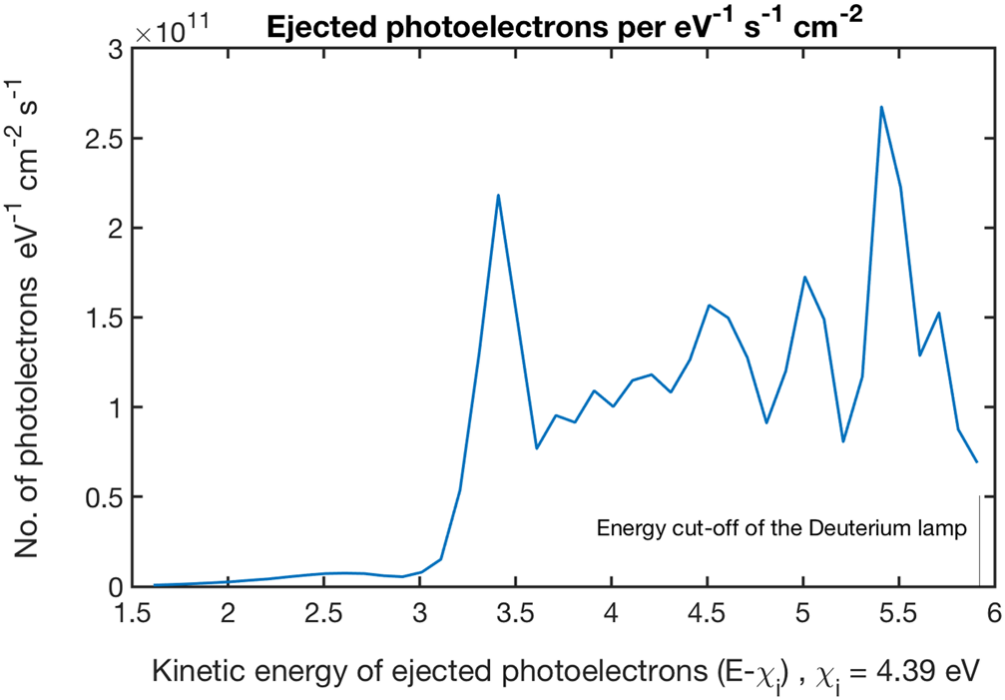
Raman spectra at several locations in the carbon fibre. The red spectrum is similar to the diamond like carbon spectrum and obtained in the main body of the fibre^19^. The black spectra have been obtained on the crystallites, at two different locations.

The observed diamondization of graphite occurs preferentially at certain locations probably tracing defects or impurities. It is most likely caused by the efficient removal of interlayer electrons by photoelectric absorption. A simple estimate based on the properties of the lamp, the irradiation time and the electric yield of graphite can be made. The samples were irradiated in a vacuum chamber using a Deuterium lamp. The lamp uses a MgF_2_ window that is transparent down to a wavelength of 115 nm corresponding to energies of 10.8 eV. For a given photon energy, the yield is defined as the number of electrons ejected from the material per absorbed photon. Graphite’s photoelectric yield is well quantified experimentally^20,21^ and these values have been used to compute the yield produced by the Deuterium lamp shown in Fig. 6; according to these calculations the number of ejected photoelectrons ranges from 8.5 × 10^10^ to 36.7 × 10^10^ photo-electrons cm^−2^ s^−1^ depending on whether the photoelectric properties of bulk graphite or those of 30 nm grains are considered. For the size of the HOPG samples (2 × 2 cm^2^) and the irradiation time (13 h), this accounts for a grand total of 1.59 × 10^16^–6.87 × 10^16^ electrons. This is a huge number equivalent to a 0.27% of the carbon atoms in a graphene sheet of 2 × 2 cm^2^.

Photoelectrons need to overcome the electric field created in the process^20–22^ however, as shown in Fig. 6, their kinetic energy is high enough for this purpose. A simple estimate of the average field can be made by noting the simple geometry of the HOPG sample; the estimated electric field is E = σ/2ε_0_ = 3.6 × 10^3^ V/m for 1.59 × 10^16^ ejected photoelectrons.

This process only occurs at the surface of the material and does not propagate significantly inwards, as shown by the STEM images. UV radiation is efficiently absorbed both by graphite and diamond. Indeed, the absorption coefficient of diamond at wavelengths shorter than 227 nm is much larger than 10^6^ cm^−1^ or 0.1 nm^−1^^23–25^; surface roughness, ^13^C isotopic fraction or impurities (diamond type) only affect slightly to this value.

Hence photoelectrons come only from the outermost graphene layers. As interlayer mobility is difficult in graphite^14,15^, the holes created by the departing electrons will be replenished by electrons from the same layer. The large number of holes created during the UV irradiation render the graphitic lattice instable by the redistribution of electrons from the π bands with bonding character.

Geometry is also relevant in the process; an increased surface curvature enhances the electric field inside the sample and moreover, the dislocation of the graphitic layers reduces very significantly the electrons mobility favouring a local growth of the electric field. This effect is neatly shown in the STEM images where the dislocation of the graphitic layers is more significant within the bumps than in the flat surface. The high mobility of the electrons within the graphite interlayers favours the appearance of large bumps once the equilibrium is broken in a given region. This results in an increasing number of bumps as the irradiation time grows, as otherwise observed.

## Materials and methods

A commercial L10366 Hamamatsu Deuterium lamp with an aperture of 0.5 mm was used as radiation source. Graphite samples were mounted at 180 mm distance and normal incidence from the deuterium lamp and then exposed to far UV radiation within a vacuum chamber at working pressures 2 × 10^–3^–7 × 10^–4^ Pa. The exposure times ranged from 2 to 13 h. The lamp uses an MgF_2_ window transparent to UV photons down to a wavelength of 115 nm.

Both HOPG samples and carbon fibers were obtained from trusted commercial providers. The size of the HOPG samples was 0.6 × 7 × 7 mm and the diameter of the optical fibers 7 μm.

For STEM, the HOPG specimens were prepared by focused ion beam (FIB) using a Dual Beam Helios Nanolab 650. EEL-line scans were performed at C K absorption edge using a Gatan Quantum EEL spectrometer in Dual EELS with an energy dispersion of 0.25 eV. EEL spectra intensity at C K line was obtained from multiple linear least-square (MLLS) fitting^26,27^ after a Fourier-Ratio Plural Scattering removal and a zero-loss centering^28^.

Raman spectra were obtained using a micro-Raman in Via Renishaw spectrograph, equipped with an electrically cooled CCD camera, and a Leica DM 2500 microscope. Laser excitation at 532 nm (Samba model, Cobolt; diffraction grating of 1800 l/mm), and 442 nm (Kimmon Koha Co., Ltd; diffraction grating of 2400 l/mm) were used as excitation sources. The laser power was 5% of the total power (1 mW at the sample) and the spectral resolution was set to 2 cm^−1^. The integration time of measurements was 10 s.
